# Personal values and involvement in problem behaviors among Bahamian early adolescents: a cross-sectional study

**DOI:** 10.1186/1471-2458-7-135

**Published:** 2007-07-02

**Authors:** Hongjie Liu, Shuli Yu, Lesley Cottrell, Sonja Lunn, Lynette Deveaux, Nanika V Brathwaite, Sharon Marshall, Xiaoming Li, Bonita Stanton

**Affiliations:** 1 The Carman and Ann Adams Department of Pediatrics, Wayne State UniversitySchool of Medicine, 4201 St. Antoine Street, UHC-6D, Detroit, Michigan 48201, USA; 2 Pediatrics, West Virginia University, Morgantown, West Virginia, USA; 3 Office of AIDS, Bahamas Ministry of Health, Nassau, Bahamas

## Abstract

**Background:**

Few studies, particularly in developing countries, have explored the relationship between adolescents and parental values with adolescent problem behaviors. The objectives of the study are to (1) describe adolescents' personal values, their problem behaviors, and the relationships thereof according to gender and (2) examine the relationship between parental values, adolescent values, and adolescents' problem behaviors among sixth-grade students and one of their parents.

**Methods:**

The data used in these analyses were from the baseline assessment of a school-based HIV risk reduction intervention being conducted and evaluated among sixth grade students and one of their parents across 9 elementary schools in The Bahamas. Personal values were measured by the Portrait Values Questionnaire (PVQ). Seven reported problem behaviors were queried from the students, which included physical fight with a friend, drank alcohol, beer, or wine, smoked a cigarette, pushed or carried any drugs, carried a gun, knife, screwdriver or cutlass to use as a weapon, had sex and used marijuana or other illicit drugs over the past 6 months. Multilevel modeling for binary data was performed to estimate the associations between adolescent and parental values and adolescent problem behaviors.

**Results:**

Among 785 students, 47% of the students reported at least one problem behavior. More boys (54%) reported having one or more problem behaviors than girls (41%, p < 0.01). Boys compared to girls expressed a higher level of self-enhancement (means score: 36.5 vs. 35.1; p = 0.03), while girls expressed a higher level of self-transcendence (42.3 vs. 40.7; p = 0.03). The results of multilevel modeling indicates that boys with a higher level of self-enhancement and girls with a higher level of openness to change and a lower level of conservation were more likely to report engagement in problem behaviors. Only two parental values (self-transcendence and conservation) were low or modestly correlated with youth' values (openness to change and self-enhancement). Parental-reported values documented limited association on adolescents' reported values and behaviors.

**Conclusion:**

In designing interventions for reducing adolescents' problem behaviors, it may be important to understand the values associated with specific problem behaviors. Further exploration regarding lack of association between adolescent and parental values and problem behaviors is needed.

## Background

Values are defined as desirable, abstract goals that motivate actions [1,2]. Schwartz's Value Theory identifies 10 basic values postulated to be universally important in societal functioning [2]: power, achievement, hedonism, stimulations, self-direction, universalism, benevolence, tradition, conformity, and security. These 10 basic human values guide the selection and evaluation of behaviors [3,4]. Bardi and Schwartz posited two explanations as to why people behave according to their values[5]: (1) there is a need for consistency between one's beliefs (values) and action; and, (2) value-consistent action is rewarding as it helps people attain satisfaction from this action. Empirical research has linked values to behaviors among adult populations; however, few studies have been conducted among adolescents. Given their developing moral reasoning and abstract thinking [6,7], adolescents may pursue different values from adults [8]. For example, adolescents prioritize values in power, achievement, self-direction and stimulation, whereas adults give priority to benevolence, universalism, tradition and conformity [7]. As a result, value-motivated behaviors may differ between adolescents and adults.

Previous studies have linked values to problem or risk behaviors [9,10]. It has been suggested that problem behaviors result in part from a deviant or oppositional self-image, which may reflect the values adopted as guiding principles [11]. For example, some research has found that the values of independence and excitement are associated with having deviant peers and substance abuse [12]. Likewise, in a sample of 544 high school students, Goff and Goddard reported students most prone to problem behaviors were those with dominant values of fun/enjoyment and security [9]. Research has documented sensation seeking, an individual intrapersonal trait, to be a major factor affecting problem or risk behaviors [13], such as drug use [14], smoking [15], alcohol use [16] and risky sexual behavior [17]. However, few studies took personal values into consideration when the association between sensation seeking and problem behaviors was examined. A well-established body of research has demonstrated that childhood experiences of problem behaviors are associated with poor mental health outcomes among adults [9,18-21]. Thus, research on the association between children's values and their problem behaviors may provide important information for development of interventions for reducing adolescents' problem behaviors.

Families, the central context for value socialization, are considered an important factor for the development of children's values [22]. However, it has been difficult to demonstrate empirically the influence of parental values on those of their offspring [23]. Previous studies have documented that parental values and children's values are poorly correlated [23-25]. Research suggests that children may or may not perceive parental values accurately; further, children may accept or reject the parental values they perceive [26,27]. Thus, the poor correlation between parental values and children's values could result from children's inaccurate perception of their parents' values or their rejection of their parental values.

Finally, in addition to the research questions remaining in this field in general, the voids are even greater among populations living in developing countries where little research has been conducted regarding youth and parent values.

In this study, we first describe adolescents' personal values and their problem behaviors according to gender and the personal values relationship to problem behaviors. We then examine whether parental values associated with adolescents' value and problem behaviors.

## Methods

The data used in these analyses were from the baseline assessment of a school-based HIV risk reduction intervention being conducted and evaluated in The Bahamas, in the autumn of 2004. In this randomized controlled intervention trial, sixth grade students and one of their parents were randomized at the level of school to receive two intervention programs: one addressing HIV/AIDS, and the other addressing ecology of the wetlands [28]. Both programs were recognized by the Department of Education as part of the sixth grade curriculum; thus all sixth grade children in the participating schools received either the ecology or the HIV prevention curriculum (according to the randomization of the school) as part of the regular school curriculum.

### Selection of schools

Only government (public) schools were included in the study. The Ministry of Education has divided the school system into districts; the island of New Providence contains four districts based on geography. Among the 26 government elementary schools in New Providence, six are in the Northwestern, five in the Northeastern, nine in the Southwestern, and six in the Southeastern Districts. Further, the schools are graded by pupil size from A to E, with A schools being the largest (approximately five sixth grade classes) and E the smallest (approximately 1 sixth grade class). In New Providence, there are 11 A schools, 7 B schools, 6 C schools, one each D and E schools. Because this is a Ministry of Education initiative, there was a desire for geographic representation. From the perspective of the research team, for purposes of adequate sample size, there was interest in participation by schools with larger student populations and for logistic reasons, participation was to be limited to 15 schools.

Prior to beginning the study, the Ministry of Education invited all sixth grade teachers and nurses from the 26 schools to a summer training which described the two curricula which were to be part of the official Ministry of Education Curriculum. The interest in obtaining appropriate geographic representation and larger school size was emphasized.

Ultimately three (50%) schools in the Northwest, three (60%) in Northeast, four (44%) in the Southwest, and five (83%) schools in the Southeast (including nine A schools, four B schools, and two C schools) sent representatives to the training and thus were included in the study. The distribution was relatively geographically equal (with slight over-representation of Southeastern Schools). Included among the fifteen schools were the three elementary schools with the highest proportion nationally of Creole-speaking families (ranging from 23 to 25% compared to an estimated 8% in New Providence). In addition to the language and some cultural barriers, the Creole population tends to have a lower socioeconomic status compared to the general Bahamian population. The most rural parts of New Providence (represented by the two D and E schools that did not send representatives to the training), were not included in this study.

### Subjects and interview

Eligible participants included all sixth-grade students (and one of their Parents) who entered the study in the first year of the study. By random selection (using a random number table), nine of the 15 school were started in the first year (2004) and six in the second year (2005) to simplify field logistics. The values questions were administered to the youth and parents in the nine schools started in the first year, but, in order to shorten the length of the questionnaire, were not included in the six schools started in the second year of the study.

Approximately two-thirds of eligible students (with one of their parents) completed and returned assent/consent forms indicating their willingness to participate. The research team administered, using a pencil and paper questionnaire, an interview to the students during class time. The questionnaire was read aloud by an interviewer to the students while they completed their questionnaire. A face-to-face interview was delivered to the parent at a mutually convenient location, generally the elementary school in the evening. Each child was given a voucher for $5 and each parent for $7 for food at a local store. The length of the questionnaire interview was approximately 45 minutes for adolescents and 30 minutes for parents. The research protocol was approved by the Institutional Review Boards at Wayne State University and Princess Margaret Hospital in Nassau, The Bahamas.

### Measures

The Bahamian Youth Health Risk Behavioral Inventory (BYHRBI), based on the Youth Health Risk Behavioral Inventory (YHRBI) [29], was used for students. An abbreviated version adapted for parents was used to assess parental perceptions. These two questionnaires had been culturally adapted for The Bahamas through extensive ethnographic research and pilot-testing [30]. The YHRBI has been used in our previous studies among students in both the US and developing countries (e.g., Bahamas, Viet Nam and China). The measures regarding children's problem behaviors were modified on the basis of the results from these previous studies and the qualitative study in the current research project. The BYHRBI assesses self-reported problem behaviors, perceptions, knowledge, and intentions to engage these behaviors.

#### Problem behaviors

Students' reported problem behaviors were measured by the following seven questions with a "Yes" or "No" answer: In the last six months, (1) did you smoke a cigarette? (2) did you drink alcohol, beer, wine rum or liquor? (3) did you push or carry any drugs? (4) did you use marijuana or any form of cocaine, including powder, crack, freebase or rock? (5) were you in a physical fight with a friend, including boyfriend or girlfriend? (6) did you carry a gun, knife, screwdriver or cutlass to use as a weapon? and (7) did you have sex? All of the seven behaviors are abnormal in school settings in The Bahamas. Based on the distribution of these problem behaviors, we dichotomized the measure into two levels: reported at least one of the 7 problem behaviors or none of them.

#### Values

Adolescent values and parental values were measured by the Portrait Values Questionnaire (PVQ)[7] The original PVQ, consisting of 40 items, measures 10 basic values. For the current study, the PVQ was modified to include 39 items (one item was deleted as it was perceived to be inappropriate in the Bahamian setting). The 10-value items were assessed by a 6-point Likert scale from 1 (not at all like me) to 6 (very much like me). According to Schwartz, the conflicts and congruities among the 10 basic values yield an integrated structure of values [31]. This structure can be summarized in two orthogonal dimensions [32]: self-enhancement vs. self-transcendence and openness to change vs. conservation. On the first dimension (self-enhancement vs. self-transcendence), power and achievement values oppose universalism and benevolence values. As postulated by Schwartz, "This dimension arrays values in terms of the extent to which they motivate people to enhance their own personal interests even at the expense of others (self-enhancement) versus to transcend selfish concerns and promote the welfare of others, close and distant and of nature (self-transcendence)" [32], page 236]. On the second dimension (openness to change vs. conservation), self-direction and stimulation values oppose security, conformity and tradition values. According to Schwartz and Boehnke [32], page 236], "The second dimension arrays values in terms of the extent to which they motivate people to follow their own emotional and intellectual interests in unpredictable and uncertain directions (openness) versus to preserve the status quo and the certainty it provides (conservation)." Hedonism shares elements of both openness and self-enhancement. On the basis of the 2 dimensions, 4 summated scores were calculated to measure the 4 values: self-enhancement (9 items; response range: 9–54), self-transcendence (10 items; response range: 10–60), openness to change (10 items, response range: 10–60) and conservation (14 items; response range: 14–78). The reliability alpha for the 4 measures in students was between 0.76–0.87 for students and between 0.74–0.80 for parents.

#### Sensation seeking

Adolescent and parental levels of sensation seeking were measured by a 4-item scale [33,34]. Respondents were asked to indicate their agreement on a 5- point Likert-type scale ranging from 1 (strongly disagree) to 5 (strongly agree) with the following items: "I would like to explore strange places"; "I like frightening things"; I like new and exciting experiences, even if I have to break the rules"; and "I prefer friends who are exciting and unpredictable." Responses to these items were then added to produce a summated score. The reliability alpha was 0.64 for the youth and 0.71 for parents.

### Analysis

In univariate analysis, student *t *test, χ^2 ^test, or Pearson's correlation (r) were used to describe the sample characters. Because this study design involved a hierarchical structure, e.g., students (level 1) nested in each selected school (level 2), multilevel modeling for binary data was performed to estimate the associations between values and problem behaviors. Intraclass correlations (ICC) of the variable of problem behaviors were estimated, which tells what portion of the total variance occurs at the second level (between schools). Intraclass correlation was 0.05. In order to assess the additive effects of adolescent and parental values on adolescent problem behaviors, we dichotomized the levels of their values into two levels based on the 75th percentile of each value distribution; high level: ≥ the 75^th ^percentile, and low level: < the 75^th ^percentile. The GLIMMIX procedure in SAS 9.1 (SAS Institute, Cary, NC) and the GLLAMM procedure in STATA 9 (StataCorp LP, College Station, TX) were used to fit multilevel models. Although all students were in the sixth grade, the ages ranged from 9–14 years because some had skipped one or more grades and others had repeated one or more grades. Students' age, a potential confounding factor, was put into the models as a covariate. These two independent procedures generated almost identical results.

## Results

### Distribution of the reported problem behaviors

In total, 785 sixth-grade students were interviewed from the nine elementary schools entering the study in the first year, with between 72 and 105 students per school. Fifty-four percent (422/785) of the students were female. The mean age of the subjects was 10.5 years old (Standard deviation [SD] = 0.73), 10.6 (SD = 0.76, range: 9–14) for boys and 10.4 (SD = 0.69, range: 9–13 for girls (t = 4.4; p < 0.01). Eighty-two percent (647/785) of the students' parents participated in the study.

Overall, 47% of the students reported at least one problem behavior (Table 1). Thirty-one percent (240) of the students reported having engaged in one problem behavior, and 16% (127) reported having multiple problem behaviors. More boys (54%) reported having one or more problem behaviors than girls (41%) (χ^2 ^= 14.2; p < 0.01). Students who engaged in at least one problem behavior were older than students who did not engage in these behaviors in the past six months (t = -3.23, p <0.01).

**Table 1:** Reported problem behaviors among the 6-grade students in the last 6 months

	**Boys (n = 363)**		**Girls (n = 422)**		
			
**Problem Behaviors**	N	%	N	%	p – value
Physical fight with a friend	154	42.5	112	26.5	< 0.01
Drank alcohol, beer, or wine	96	26.5	92	21.8	0.15
Smoked a cigarette	9	2.5	10	2.4	0.90
Pushed or carried any drugs	10	2.8	0	0	< 0.01
Carried a gun, knife, screwdriver or cutlass to use as a weapon	26	7.2	3	0.7	< 0.01
Had sex	8	2.2	2	0.5	0.03
Used marijuana or other illicit drugs	7	1.9	2	0.5	0.06

### Distributions of values and sensation seeking

Table 2 depicts the four measures of personal values and sensation seeking. Boys compared to girls expressed a higher level of self-enhancement, while girls expressed a higher level of self-transcendence. Boys expressed a higher sensation seeking desire than did girls. The levels of values in openness to change and conservation did not statistically differ between boys and girls.

**Table 2:** Distribution of values and sensation among boys and girls

	**Boys (n = 363)**	**Girls (n = 422)**	
			
**Values and sensation seeking**	Mean (SD)	Mean (SD)	p – value
Self-enhancement	36.5 (8.85)	35.1 (9.00)	0.03
Self-transcendence	40.7 (10.47)	42.28 (10.56)	0.03
Openness to change	41.4 (9.74)	40.4 (9.91)	0.16
Conservation	54.7 (13.32)	56.5 (13.14)	0.06
Sensation seeking	9.3 (3.78)	7.6 (3.79)	< 0.01

SD: Standard Deviation

### Associations between youth values, sensation seeking and problem behaviors

The results of multilevel modeling indicate that boys with a higher level of self-enhancement and girls with a higher level of openness to change and a lower level of conservation were more likely to report problem behaviors. Sensation seeking was not statistically associated with the reported problem behaviors (Table 3).

**Table 3:** Associations between values, sensation and problem behaviors

	**Boys (n = 363)**		**Girls (n = 422)**	
		
**Values and sensation seeking**	OR	95 % CI	OR	95% CI
Self-enhancement	**1.38**	**1.10–1.75**	0.99	0.80–1.21
Self-transcendence	0.85	0.68–1.05	1.18	0.95–1.42
Openness to change	0.98	0.78–1.23	**1.34**	**1.10–1.68**
Conservation	0.84	0.70–1.02	**0.74**	**0.62–0.89**
Sensation seeking	1.24	0.92–1.68	1.23	0.93–1.62

OR: odds ratio

95% CI: 95% confidence interval

### Associations between additive levels of parental and adolescents' values, sensation seeking and student's problem behaviors

Pearson's correlation analysis was performed among adolescent and parental values and sensation seeking. The results document that for only two values were parental values significantly correlated with youth' values; for both of these values the correlation was at a relatively low level (the range of the significant correlation coefficients was between -0.14 – 0.12). Specifically, parental self-transcendence was positively correlated with boys' openness to change (r = 0.12, p = 0.03)) and negatively with girls' self-enhancement (r = -0.11, p = 0.03) and openness to change values (r = -0.11, p = 0.05). Parental conservation was negatively correlated with girls' self-enhancement (r = -0.14, p = 0.01).

We sought to determine if there was an additive effect of shared values between parents and youth on the selected problem behaviors. Table 4 shows mixed patterns of the associations between additive levels of adolescent and parental values and sensation seeking on adolescents' problem behaviors. Only the additive levels of sensation seeking was positively associated with boys' problem behaviors.

**Table 4:** The relations between problem behaviors and additive level of child's and parents' values and sensation

			**Boys (n = 309)**		**Girls (n = 338)**	
				
**Values and sensation seeking**	**Child**	**Parents**	OR	95% CI	OR	95% CI
Self-enhancement	L	L	1		1	
	L	H	**2.20**	**1.08–4.14**	1.43	0.78–2.62
	H	L	0.79	0.43–1.47	0.89	0.49–1.63
	H	H	1.24	0.59–2.60	1.06	0.37–0.92
Self-transcendence	L	L	1		1	
	L	H	**2.20**	**1.17–4.12**	0.97	0.51–1.86
	H	L	0.58	0.30–1.12	1.11	0.62–2.01
	H	H	0.62	0.23–1.68	1.78	0.85–3.68
Openness to change						
	L	L	1		1	
	L	H	**1.91**	**1.00–3.66**	1.40	0.74–2.63
	H	L	0.72	0.38–1.36	1.67	0.93–3.02
	H	H	0.98	0.44–2.17	2.42	0.96–6.13
Conservation						
	L	L	1		1	
	L	H	1.42	0.76–2.65	1.16	0.63–2.13
	H	L	0.55	0.29–1.07	1.03	0.58–1.85
	H	H	0.47	0.19–1.13	1.27	0.54–2.96
Sensation seeking						
	L	L	1		1	
	L	H	2.07	0.98–4.35	1.56	0.87–2.80
	H	L	1.37	0.78–2.37	1.50	0.80–2.81
	H	H	**2.69**	**1.13–6.41**	0.97	0.38–3.44

H: high level; equal to or higher than 75th percentile

L: low level: lower than 75th percentile

OR: odds ratio

95% CI: 95% confidence interval

## Discussion

The findings from this study document that adherence to specific values is associated with involvement in problem behaviors among Bahamian adolescents. Consistent with the published literature among older individuals [3,35], these associations differ by gender, suggesting that female and male students may share different value-behavior mechanisms. Parental-reported values demonstrated limited associations on adolescents' reported values and behaviors.

Values express what is important to adolescents in their lives [5]. Gender may influence the importance of specific values. It has been postulated that men emphasize agentic-instrumental values such as power, whereas women emphasize expressive-communal values such as benevolence [36]. Our study found that boys gave higher priority than girls to self-enhancement values, whereas girls prioritize self-transcendence values. These findings are similar to those from studies conducted in the US, Australia and nine East Asian countries [3,35].

These findings are consistent with psychoanalytic theory and social role theory [36-38]. According to psychoanalytic theory, women are more related and more affiliated with others than men, whereas men are more autonomous and more individuated [38]. Social role theorists contend that women migrate to nurturing roles which reduces competition and preserves harmonious relationships, whereas men tend to engage in more instrumental, task-oriented roles [37,39].

Because societies hold different expectations regarding gender in terms of social status and roles [40-42], males and females are socialized to attain different values and to occupy different social roles. Consistent with this perspective, males engaged in more problem behaviors than females. Possibly, engaging in these problem behaviors, boys may feel that they are demonstrating their competence in social power, authority or achievement to their peers [42]. The higher level of sensation seeking in boys indicates that boys' involvement in problem behaviors may result from the pursuit of pleasure, excitement, and/or novelty. Research has documented that younger people report higher values in self-enhancement while older people report higher values in self-transcendence [7]. As they age, adolescents' values become more realistic [43].

Values express basic human needs [1,44], and these needs motivate behaviors [6]. Individual differences in value priorities have been reported to relate meaningfully to actual behaviors such as prosocial, antisocial, environmental, political, consumer, and delinquent behaviors [5,31,45]. While research supports the relationship between adult values and behaviors [6,7], few studies have focused on the linkage among early adolescents. The findings from the current study revealed that some values were associated with problem behaviors among early adolescents. Specifically, boys with a higher level of self-enhancement values were more likely to engage in problem behaviors, whereas, girls with a higher level of openness to change and a lower level in conservation were more likely to engage in problem behaviors. These findings may be reflected in the theory proposed by Schwartz and colleagues [7], which speculates that attributing an extremely high priority to a value induces overly intense and rigid pursuit of that value. For example, people who value self-enhancement and openness to change are more likely to pursue their own success and dominance over others in an exaggerated manner, or to emphasize their own independent thought and experience risk taking and adventure [7,45]. By contrast, people who attribute little importance to the above two values or who endorse high importance to opposed values are less likely to exhibit these behavioral patterns. For example, people who highly rate conservatism may seek to preserve traditional practices, and protect stability. Thus, they may be less likely to engage in problem behaviors. Boys engaging in problem behaviors prioritized self-enhancement values, whereas, girls engaging in problem behaviors placed a high value on openness to change, suggesting a difference in motivational goals [46,47]. Empirical research has demonstrated that behaviors can be changed by changing the core values which motivate behaviors [1,48]. Thus, interventions targeting reduction of students' problem behaviors should take into consideration the gender difference in the value-inducing problem behaviors.

Parental values were generally not correlated with adolescent reported values and there were minimal additive effects of parental and youth values and sensation seeking on adolescents' reported problem behaviors. Our results corroborate previous research that documents the low-to-modest degree of correlations between parental values and children's values [23,24], and expand upon this literature by including youth and parents from a developing country since most prior research in this area had been conducted in developed country settings. This lack of correlation may be explained by the two-process model proposed by Grusec and Goodnow [26]. According to this model, children first perceive parents' values either accurately or inaccurately. Subsequently, children decide whether they accept or reject parental values largely based on their perceptions of parental values. Research has documented that the relationship between parents' actual values and their children's actual values is mediated by the children's perceptions of their parents' values [49]. If children's perceptions of their parents' values are incorrect, children may not correctly follow their parents' values. As a result, adolescent values may not be associated with parental actual values reported by parents.

Several researchers have argued that it is children's perceptions of parents' beliefs or values, and not parents' actual (i.e., self-reported) beliefs or values, that are predictive of children's values [50-52]. Cashmore and Goodnow found high perceived agreement between parents and adolescents but low actual agreement [53]. Daughters, compared to sons, may have relatively more accurate perceptions of their parents' values. For example, daughters' self-enhancement values negatively correlated with their parents' self-transcendence and conservation values, and daughters' openness to change values were negatively associated with parents' self-transcendence values. However, these correlations were at a fairly low level and may not be manifest as a significant influence on adolescents' problem behaviors. While the two-process model may be applicable to the explanation of the finding, further research is needed to explore other possible reasons on the non-associations.

Several limitations to this study need to be noted. Because the data are cross-sectional, results should be interpreted as associations which do not imply causality. Only nine schools were selected to this study and two thirds students in the selected schools participated. We were not able to assess if the students participating in the study represented the wider population of all students at the age in The Bahamas. Because responses were assessed retrospectively, recall bias may be present. Some problem behaviors are not socially acceptable; study participants may therefore have provided socially desirable responses. Further studies are needed to validate questions regarding problem behaviors. Although the scale for sensation-seeking has been widely used by researchers, the reliability and validity of the scale should been further examined and improved. In addition, we were unable to examine whether parental socio-economic status, e.g., education and religiosity, were associated with their child's values because information on SES was not queried in the questionnaire survey. This association needs to be examined in future studies.

## Conclusion

Despite the study's limitations, the findings may have some significant implications for interventions designed to reduce problem behaviors among students. Interventions need to take into account the specific values that motivate students' problem behaviors. Values self-confrontation methods, which were pioneered by Rokeach [1], seek to change people's behaviors by changing the priority of the values that motivate the behaviors. Values self-confrontation has been successfully used to reduce smoking [54] and increase weight loss [55]. Further research should be conducted to determine its effectiveness with adolescents' problem behaviors.

As males and females share different value-behavior mechanisms, interventions should consider gender differences in the value-induced problem behaviors. Likewise, interventions at the family level should not be predicated only on parents' values and value-induced beliefs may not be consistent with their children's values. Future research on value modification and transmission between parents and children is needed to identify the effect ways in which children learn about their parents' values, and to explore intervention strategies which can be used to influence children's values and behaviors by their parents' values.

## Competing interests

The author(s) declare that they have no competing interests.

## Authors' contributions

HJ, SY, LC, SM, XL and BS participated in the design of the study, carried out the quality control, performed the statistical analysis and drafted the manuscript. SL, LD and NVB involved in the design of the study, implemented study procedures and acquired of data. All authors read and approved the final manuscript.

## Pre-publication history

The pre-publication history for this paper can be accessed here:



## References

[B1] Rokeach M (1973). The nature of human values.

[B2] Schwartz SH, Zanna MP (1992). Universals in the content and structure of values: Theoretical advances and empirical tests in 20 countries. Advances in experimental social psychology.

[B3] Beutel AM, Marini MM (1995). Gender and values. American Sociological Review.

[B4] Maio GR, Olson JM (1995). Relations between values, attitudes, and behavioral intentions: The moderating role of attitude function. Journal of Experimental Social Psychology.

[B5] Bardi A, Schwartz SH (2003). Values and Behavior: Strength and Structure of Relations. Personality and Social Psychology Bulletin.

[B6] Hitlin S, Piliavin JA (2004). Values: Reviving a dormant concept. Annual Review of Sociology.

[B7] Schwartz SH, Melech G, Lehmann A, Burgess S, Harris M, Owens VE (2001). Extending the cross-cultural validity of the theory of basic human values with a different method of measurement. Journal of Cross-Cultural Psychology.

[B8] Smith PB, Peterson MF, Schwartz SH, Ahmad A (2002). Cultural values, sources of guidance, and their relevance to managerial behavior: A 47-nation study. Journal of Cross-Cultural Psychology.

[B9] Goff BG, Goddard HW (1999). Terminal core values associated with adolescent problem behaviors. Adolescence.

[B10] Chernoff RA, Davison GC (1999). Values and their relationship to HIV/AIDS risk behavior among late-adolescent and young adult college students. Cognitive Therapy and Research.

[B11] Grube JW, Mayton DM, Ball-Rokeach SJ (1994). Inducing change in values, attitudes, and behaviors: Belief system theory and the method of value self-confrontation. Journal of Social Issues.

[B12] Simons RL, Whitbeck LB, Conger RD, Melby JN (1991). The effect of social skills, values, peers, and depression on adolescent substance use. Journal of Early Adolescence.

[B13] Zuckerman M (1994). Behavioral expressions and biosocial bases of sensation seeking.

[B14] Yanovitzky I (2005). Sensation Seeking and Adolescent Drug Use: The Mediating Role of Association With Deviant Peers and Pro-Drug Discussions. Health Communication.

[B15] Zuckerman M, Ball SA, Black J (1990). Influences of sensation seeking, gender, risk appraisal, and situational motivation on smoking. Addictive Behaviors.

[B16] Donohew RL, Hoyle RH, Clayton RR, Skinner WF, Colon SE, Rice RE (1999). Sensation seeking and drug use by adolescents and their friends: Models for marijuana and alcohol. Journal of Studies on Alcohol.

[B17] Sheer VC, Cline RJ (1994). The development and validation of a model explaining sexual behavior among college students: Implications for AIDS communication campaigns. Human Communication Research.

[B18] Guilamo-Ramos V, Litardo HA, Jaccard J (2005). Prevention programs for reducing adolescent problem behaviors: Implications of the co-occurrence of problem behaviors in adolescence. Journal of Adolescent Health.

[B19] Arthur MW, Hawkins JD, Pollard JA, Catalano RF, Baglioni AJ (2002). Measuring risk and protective factors for substance use, delinquency, and other adolescent problem behaviors. The Communities That Care Youth Survey. Evaluation Review.

[B20] Raboteg-Saric Z, Rijavec M, Brajsa-Zganec A (2001). The relation of parental practices and self-conceptions to young adolescent problem behaviors and substance use. Nordic Journal of Psychiatry.

[B21] Bensley LS, Van Eenwyk J, Spieker SJ, Schoder J (1999). Self-reported abuse history and adolescent problem behaviors. I. Antisocial and suicidal behaviors. Journal of Adolescent Health.

[B22] Roberts REL, Bengtson VL, Ryff C, Marshall VW (1999). The social psychology of values: Effects of individual development, social change, and family transmission over the life span. The self and social processes in aging.

[B23] Whitbeck LB, Gecas V (1988). Value attributions and value transmission between parents and children. Journal of Marriage & the Family.

[B24] Thomas LE, Stankiewicz JF (1974). Family correlates of parent-child attitude congruence: Is it time to throw in the towel?. Psychological Reports.

[B25] Connell RW (1972). Political socialization in the American family: The evidence re-examined. Public Opinion Quarterly Vol 36(3).

[B26] Grusec JE, Goodnow JJ (1994). Impact of parental discipline methods on the child's internalization of values: A reconceptualization of current points of view. Developmental Psychology.

[B27] Knafo A, Schwartz SH (2003). Parenting and adolescents' accuracy in perceiving parental values. Child Development.

[B28] Deveaux L, Stanton B, Lunn S, Yu S, Brathwaite N, Li X, Liu H, Marshall S, Harris C, Cottrell L (2007). Adolescent risk reduction in developing countries: an HIV prevention intervention with and without a parental monitoring component targeting sixth grade students in The Bahamas. Archives of Pediatrics & Adolescent Medicine.

[B29] Stanton B, Black M, Feigelman S, Ricardo I, Galbraith J, Li X, Kaljee L, Keane V, Nesbitt R (1995). Development of a culturally, theoretically and developmentally based survey instrument for assessing risk behaviors among African-American early adolescents living in urban low-income neighborhoods. AIDS Educ Prev.

[B30] Yu S, Deveaux L, Clemens R, Cottrell L, Harris C, Lunn S, Yang H, Li X, Stanton B (2006). Youth and Parental Perceptions of Parental Monitoring and Parent-adolescent Communication Among Depressed and Non-depressed Pre-adolescent Youth. Social Behavior and Personality: An International Journal (in press).

[B31] Schwartz SH, Bardi A (2001). Value hierarchies across cultures: Taking a similarities perspective. Journal of Cross-Cultural Psychology.

[B32] Schwartz SH, Boehnke K (2004). Evaluating the structure of human values with confirmatory factor analysis. Journal of Research in Personality.

[B33] Hoyle RH, Stephenson MT, Palmgreen P, Pugzles Lorch E, Donohew RLE (2002). Reliability and validity of a brief measure of sensation seeking. Personality and Individual Differences.

[B34] Yanovitzky I (2006). Sensation Seeking and Alcohol Use by College Students: Examining Multiple Pathways of Effects. Journal of Health Communication.

[B35] Schwartz SH, Rubel T (2005). Sex differences in value priorities: Cross-cultural and multimethod studies. Journal of Personality and Social Psychology.

[B36] Prince-Gibson E, Schwartz SH (1998). Values priorities and gender. Social Psychology Quarterly.

[B37] Parsons T, Bales R (1955). Family Socialization and Interaction Process.

[B38] Chodorow N, Rhode D (1990). What is the relation between psychoanalytic feminism and the psychoanalytic psychology of women?. Theoretical Perspectives on Sexual Difference.

[B39] Bakan D (1966). The duality of human existence.

[B40] Trivers RL, Campbell B (1972). Parental investment and sexual selection. Sexual selection and the descent of man: 1871-1971.

[B41] Daly M, Wilson M (1983). Sex, evolution, and behavior.

[B42] Wilson M, Daly M (1985). Competitiveness, risk taking, and violence: The young male syndrome. Ethology and Sociobiology.

[B43] Johnson MK (2002). Social origins, adolescent experiences, and work value trajectories during the transition to adulthood. Social Forces.

[B44] Schwartz SH, Sagiv L (1995). Identifying culture-specifics in the content and structure of values. Journal of Cross-Cultural Psychology.

[B45] Saroglou V, Delpierre V, Dernelle R (2004). Values and religiosity: A meta-analysis of studies using Schwartz's model. Personality and Individual Differences.

[B46] Karp DR, Borgatta EF, Montgomery RJV (2000). Values theory and research. Encyclopedia of sociolog.

[B47] Feather NT, Halisch F, Kuhl J (1987). Gender differences in values: Implications of the expectancy-value model. Motivation, intention, and volition.

[B48] Ball-Rokeach S, Rokeach M, Grube J (1984). The great American values test.

[B49] Okagaki L, Bevis C (1999). Transmission of religious values: Relations between parents' and daughters' beliefs. Journal of Genetic Psychology.

[B50] Acock AC, Bengtson VL (1980). Socialization and attribution processes: Actual versus perceived similarity among parents and youth. Journal of Marriage & the Family.

[B51] Smith TE (1982). The case for parental transmission of educational goals: The importance of accurate offspring predictions. Journal of Marriage & the Family.

[B52] Kerckhoff AC, Huff JL (1974). Parental influence on educational goals. Sociometry.

[B53] Cashmore JA, Goodnow JJ (1985). Agreement between generations: A two-process approach. Child Development Family development.

[B54] Conroy WJ, Rokeach M (1979). Human Values, Smoking Behavior, and Public Health Programs. Understanding human values : individual and societal.

[B55] Schwartz SHHUJI, Inbar-Saban N (1988). Value self-confrontation as a method to aid in weight loss. Journal of Personality and Social Psychology.

